# Genetic Diversity of *Apis cerana cerana* in the Lüliang Mountain Area Based on Molecular Markers

**DOI:** 10.3390/genes16121420

**Published:** 2025-11-28

**Authors:** Chang Song, Ke Sun, Yanting Song, Qiyan Su, Xueyan Yi, Lina Guo, Yuan Guo

**Affiliations:** 1 College of Animal Science, Shanxi Agricultural University, Taigu 030801, China; 202420362@stu.sxau.edu.cn (C.S.); 202430646@stu.sxau.edu.cn (K.S.); 202430644@stu.sxau.edu.cn (Y.S.); suqiyan@sxau.edu.cn (Q.S.); yixueyan@sxau.edu.cn (X.Y.); 2 Shanxi Key Laboratory of Animal Genetic Resource Utilization and Breeding, Taigu 030801, China; 3 College of Horticulture, Shanxi Agricultural University, Taigu 030801, China

**Keywords:** *Apis cerana cerana*, genetic diversity, Lüliang Mountains, microsatellite markers, mitochondrial DNA

## Abstract

Objectives: This study presents a comprehensive molecular investigation of *Apis cerana cerana* populations inhabiting the Lüliang Mountain region, aiming to evaluate their genetic diversity and population structure using polymorphic microsatellite and mitochondrial DNA (mtDNA) markers. Methods: A total of 23 microsatellite loci and three mtDNA fragments (*COI–COII*, *COI*, *Cytb*) were successfully amplified, of which 21 loci were polymorphic and used for subsequent genetic analyses. Measures of genetic variability, population differentiation, and molecular variance were computed to assess intra- and interpopulation diversity. Results: High levels of genetic variation were detected (mean PIC = 0.349), with observed heterozygosity (Ho = 0.827) exceeding expected heterozygosity (He = 0.608). Analysis of molecular variance (AMOVA) revealed that 95.28% of total genetic variation occurred within populations, while 4.72% was attributed to among-population differences. Mitochondrial analyses identified 20 polymorphic sites forming 19 haplotypes, with high haplotype (Hd = 0.884) and nucleotide diversity (π = 0.00157). Conclusions: These results indicate substantial gene flow and interpopulation connectivity among *A. c. cerana* populations in the Lüliang region. Collectively, the findings provide critical molecular evidence supporting the conservation and sustainable management of *A. c. cerana* genetic resources in this area.

## 1. Introduction

*A. c. cerana*, the eastern honey bee, is a key pollinator across East Asia. Through long-term natural selection, this subspecies has adapted to diverse climatic and floral conditions, contributing substantially to ecosystem stability and agricultural productivity. Genetic diversity is a fundamental determinant of colony fitness and resilience to environmental stressors, including pathogens, pesticides, and climatic fluctuations. However, over recent decades, *A. c. cerana* populations have declined due to habitat fragmentation, pesticide exposure, interspecific competition from *Apis mellifera*, and climate change [1,2]. These pressures underscore the need for robust, population-level genetic assessments to inform conservation [3].

The Lüliang Mountains, located on the eastern edge of the Loess Plateau, serve as an important ecological barrier in northern China. The area is characterized by complex terrain and diverse vegetation types ranging from temperate deciduous forests to shrub–grassland mosaics [4,5]. The continuous availability of nectar sources—such as forsythia in spring, black locust in summer, and sea buckthorn in autumn—provides favorable conditions for the long-term survival and reproduction of *A. c. cerana*. Owing to prolonged geographical isolation and local adaptation, populations from this region may possess distinct genetic features that differ from those of other areas, making them valuable material for studying population differentiation and adaptive evolution. Nevertheless, the level of genetic diversity, population structure, and maternal lineage composition of *A. c. cerana* in the Lüliang Mountains remains poorly understood.

Molecular markers have become powerful tools for investigating genetic variation and population differentiation in honeybees. Among them, microsatellite markers (simple sequence repeats, SSRs) are widely used due to their high polymorphism, co-dominant inheritance, and genome-wide distribution, making them ideal for assessing gene flow, genetic differentiation, and population structure [6]. Mitochondrial DNA (mtDNA), which is maternally inherited and exhibits a relatively high mutation rate, complements nuclear markers and provides insights into evolutionary history and maternal lineages [7].

In this study, both microsatellite and mtDNA markers were employed to systematically assess the genetic diversity and differentiation of *A. c. cerana* populations from six sampling sites across the Lüliang Mountains. The results will provide a molecular basis for understanding the evolutionary characteristics of local populations and support the formulation of strategies for conservation, breeding, and sustainable utilization of *A. c. cerana* genetic resources.

## 2. Materials and Methods

### 2.1. Sampling Strategy and Study Sites

In May 2023, worker bees of *A. c. cerana* were collected from six counties in the Lüliang Mountain region: Suide (SD; 37°30′13″ N, 110°37′14″ E), Wubu (WB; 37°39′19″ N, 110°36′47″ E), Mizhi (MZ; 37°53′03″ N, 110°05′42″ E), Zizhou (ZZ; 37°36′06″ N, 110°03′52″ E), Qingjian (QJ; 37°02′09″ N, 110°04′38″ E), and Shilou (SL; 37°01′20″ N, 110°45′57″ E). From each county, ten colonies maintained by local apiarists were selected (*n* = 60 colonies). Ninety workers were collected per colony (totaling 540 bees) and used for morphometric measurements. All specimens were preserved in 75% ethanol at −20 °C until DNA extraction.

### 2.2. Ethical Considerations

All sampling was performed with the consent of local beekeepers and complied with institutional and national guidelines for the care and use of invertebrates in research. No endangered species were involved, and no protected areas were sampled.

### 2.3. Capillary Electrophoresis Sequencing

Genomic DNA was extracted using the Tiangen Biotech DNA Extraction Kit (Beijing, China) following the manufacturer’s protocol. Twenty-three microsatellite primer pairs were screened; two loci (SV220, AP042) were monomorphic in our dataset and excluded from polymorphism-based analyses. PCR amplifications were carried out in 20 μL reactions containing 2 μL DNA template, 0.8 μL of each primer, 10 μL TB Green^®^ Premix Ex Taq™ II (Takara, Japan), and nuclease-free water. The thermal cycling profile comprised an initial denaturation at 95 °C for 2 min; 35 cycles of 95 °C for 20 s, 56–58 °C for 20 s, and 72 °C for 2 min; followed by a final hold at 4 °C. Fragment analysis was performed on an ABI 3730XL DNA Analyzer (Applied Biosystems, Foster City, CA, USA). Primer information is listed in Table 1.

### 2.4. Mitochondrial DNA Sequencing Method

Genomic DNA was extracted using the Tiangen DNA extraction kit, and PCR amplification was carried out with the TaKaRa PCR amplification kit. PCR amplifications were carried out in 20 μL reactions containing 0.5 μL DNA template, 0.5 μL of each primer, 10 μL Taq enzyme, and nuclease-free water. The polymerase chain reaction (PCR) amplification comprised an initial pre-denaturation step at 94 °C for 2 min to ensure complete DNA strand separation, followed by a denaturation step at 94 °C for 30 s to disrupt hydrogen bonds between DNA strands. The annealing step was performed at 56–58 °C for 30 s, allowing primer hybridization to target sequences, and an extension step at 72 °C for 30 s facilitated nucleotide elongation by Taq DNA polymerase. The amplification cycle was repeated 30 times to achieve sufficient DNA yield. A final extension at 72 °C for 2 min ensured complete synthesis of all DNA fragments, and the reaction was then held at 4 °C for storage prior to electrophoretic analysis. All procedures were conducted according to the manufacturer’s instructions. Primer information is provided in Table 2.

The PCR amplicons were submitted to Tsingke Biotechnology Co., Ltd. (Anhui, China) for bidirectional Sanger sequencing. Each sequencing reaction (15 µL total volume) contained 2 µL of BigDye™ Terminator v3.1 Ready Reaction Mix, 1 µL of each primer (forward and reverse), 3 µL of RNase-free H_2_O, and 15 µL of DNA template.

Thermal cycling consisted of an initial pre-denaturation at 96 °C for 1 min, followed by 25 cycles of denaturation at 96 °C for 10 s, annealing at 50 °C for 5 s, and extension at 60 °C for 4 min, with a final hold at 4 °C. All reactions were performed in the dark to prevent fluorophore degradation.

Following amplification, purification was conducted according to the system using 90 µL of SAM™ Solution and 20 µL of BigDye XTerminator™ Solution to remove residual dye terminators and salts. After brief centrifugation, the purified sequencing products were analyzed via capillary electrophoresis on an ABI 3730XL DNA Analyzer (Applied Biosystems, USA).

### 2.5. Mitochondrial DNA Amplification and Sequencing

Three mtDNA regions (*COI–COII* intergenic region, *COI*, and *Cytb*) were amplified using published primer sets. PCR products were purified and Sanger sequenced by Tsingke Biotechnology Co., Ltd. (Anhui, China). Sequences were aligned and concatenated in MEGA 11.0; polymorphism indices—including the number of variable sites (S), haplotype diversity (Hd), average number of nucleotide differences (K), and nucleotide diversity (π)—were estimated using DnaSP v6.

### 2.6. Data Processing and Statistical Analyses

Microsatellite genotypes were scored and quality-checked. Locus-wise polymorphism information content (PIC), observed allele number (Na), effective allele number (Ne), observed heterozygosity (Ho), expected heterozygosity (He), and Nei’s gene diversity (H) were computed in GenAlEx 6.51b2 and POPGENE 21.0. Deviations from Hardy–Weinberg equilibrium (HWE) and linkage disequilibrium (LD) were assessed using exact tests with Bonferroni correction. Population structure was evaluated via analysis of molecular variance (AMOVA), principal coordinate analysis (PCoA), and clustering based on genetic similarity coefficients (NTSYS-pc). Haplotype counts, diversity, and nucleotide diversity from mtDNA data were calculated with DnaSP v6.

## 3. Results

### 3.1. Genetic Diversity Based on Microsatellite Marker

**Figure 1:**
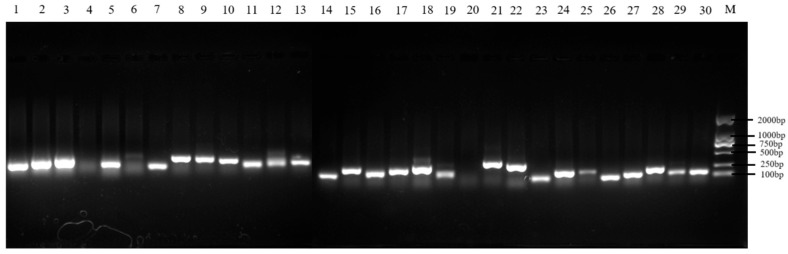
Verification by agarose gel electrophoresis. Note: Sites 1-30 represent BI216, BI278, AP249, SV220, AP066vSV261, AT185, BI225, AP208, AC139, AT103, K0715, AP148, SV066, AT165, AP042, AT109, AT101, UN117, K1458, UNEV2, UN270, SV039, Ap313, AT004, AC045, AC011, AP189, BI314, Ap085 and AP243, M represents Marker.

A total of 30 microsatellite loci were initially tested across 60 colonies of *A. c. cerana* from six sampling sites using agarose gel electrophoresis. Loci showing clear, single, and bright bands were selected for large-scale amplification and genetic diversity analysis. Ultimately, 23 pairs of microsatellite loci (BI216, BI278, AP249, SV220, AP066, SV261, AT185, BI225, AP208, AC139, AT103, K0715, AP148, SV066, AT165, AP042, AT109, AT101, UN117, K1458, UNEV2, UN270, SV039) were retained for subsequent analyses (Figure 1).

### 3.2. Microsatellite Locus Genotyping Results

**Figure 2:**
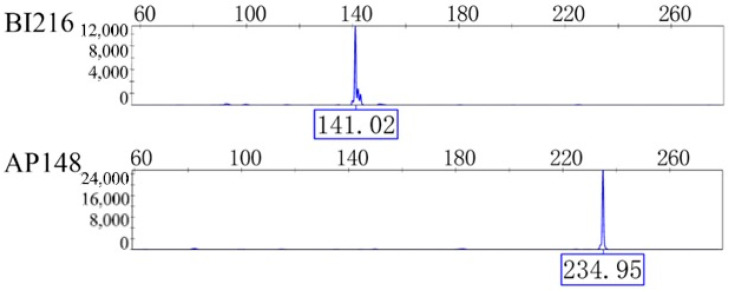
Two loci typing results of *A. c. cerana* microsatellites.

The data obtained from the 23 selected primer pairs were organized based on peak profiles, and representative electropherograms of microsatellite genotypes are presented in Figure 2.

### 3.3. Population Genetic Diversity Analysis Based on Microsatellite Markers

The polymorphism information content (PIC) values of the 23 microsatellite loci were as follows: BI216 (0.398), BI278 (0.610), AP249 (0.580), SV220 (0), AP066 (0.304), SV261 (0.362), AT185 (0.614), BI225 (0.630), AP208 (0.362), AC139 (0.733), AT103 (0.220), K0715 (0.315), AP148 (0.304), SV066 (0.222), AT165 (0.032), AP042 (0), AT109 (0.184), AT101 (0.646), UN117 (0.204), K1458 (0.121), UNEV2 (0.383), UN270 (0.247), and SV039 (0.556).

Genetic diversity indices of *A. c. cerana* populations from six sampling sites in the Lüliang Mountain region were calculated using GenAlEx 6.51b2 and POPGENE 21.0 (Table 3). Across all populations, the mean PIC was 0.349, the observed number of alleles (Na) was 2.630, the effective number of alleles (Ne) was 1.823, Nei’s gene diversity index (H) was 0.388, and Shannon’s information index (I) was 0.608. Observed heterozygosity (Ho) and expected heterozygosity (He) were 0.827 and 0.608, respectively.

At the population level, PIC ranged from 0.265 to 0.341, Na from 2.304 to 2.913, Ne from 1.689 to 1.907, H from 0.303 to 0.394, and I from 0.523 to 0.677. Ho ranged from 0.552 to 0.852, while He varied between 0.585 and 0.680. Among the six sampling sites, Qingjian exhibited the highest PIC (0.341), Ne (2.913), and H (0.394). Wubu displayed the lowest I (0.523). Mizhi had the highest Ho (0.852) and He (0.680), but also showed the lowest PIC (0.265), Na (2.304), Ne (1.689), H (0.303), and I (0.523). In contrast, Wubu had the lowest Ho (0.734), and Qingjian had the lowest He (0.585).

Overall, comparative analysis of PIC, Ne, H, and I revealed the following ranking of genetic diversity among the six populations: Qingjian > Wubu > Shilou > Suide > Zizhou > Mizhi. For all populations combined and for each individual population, observed heterozygosity consistently exceeded expected heterozygosity, indicating excess heterozygosity in these populations.

### 3.4. Genetic Differentiation Analysis Based on Microsatellite Markers

Population genetic differentiation was evaluated using hierarchical F-statistics and AMOVA. Across all sampling sites, observed heterozygosity consistently exceeded expected heterozygosity (Ho > He), yielding a negative within-population inbreeding coefficient (FIS = −0.36). This pattern reflects heterozygote excess rather than inbreeding and is consistent with the diversity indices presented in Table 3. AMOVA partitioned the genetic variation into within- and among-population components, revealing that 95.28% of total variation occurred within populations and only 4.72% among populations (*p* < 0.05). This distribution corresponds to an FST value of 0.047, indicating low-to-moderate population differentiation. After correcting the previously misreported value of FST = 0.180, the recalculated statistic is fully consistent with the observed allele frequency patterns. Gene flow estimates further clarified the population structure. The calculated Nm value of 2.74 (>1) suggests that gene exchange among sites is sufficiently frequent to counteract genetic drift, promoting genetic homogenization across the region while still allowing subtle local differentiation. The overall inbreeding coefficient (FIT = 0.533) reflects population subdivision (Wahlund effect) instead of true biological inbreeding. All F-statistics were reanalyzed under corrected settings in GenAlEx 6.51b2 to ensure statistical accuracy.

According to the AMOVA results in Table 4, 4.72% of the total genetic variation was distributed among populations, whereas 95.28% was observed within populations. This demonstrates that the majority of genetic variation resides within populations rather than among them. Such a pattern is consistent with the biological characteristics of honeybee populations, where frequent gene flow, colony migration, and mating behavior promote high levels of within-population variability while limiting genetic divergence between populations.

### 3.5. Genetic Distance and Genetic Similarity Based on Microsatellite Markers

Genetic distance and genetic similarity among populations were calculated using GenAlEx 6.51b2, and the results are presented in Table 5. The average genetic distance among the six *A. c. cerana* sampling sites was 0.075, with an average genetic similarity of 0.927. The genetic distance values ranged from 0.050 to 0.129. The greatest genetic distance was observed between Suide (SD) and Zizhou (ZZ) populations (0.129), followed by Suide (SD) and Qingjian (QJ) (0.095). In contrast, the shortest genetic distance was detected between Wubu (WB) and Mizhi (MZ) (0.050), followed by Qingjian (QJ) and Shilou (SL) (0.052).

Genetic similarity values ranged from 0.878 to 0.950. The highest similarity was observed between Wubu (WB) and Mizhi (MZ) (0.950), followed by Qingjian (QJ) and Shilou (SL) (0.948). Conversely, the lowest similarity was found between Suide (SD) and Zizhou (ZZ) (0.878), followed by Suide (SD) and Shilou (SL) (0.908).

Overall, these results indicate that Suide and Zizhou populations are the most genetically divergent, whereas Wubu and Mizhi populations are the most genetically similar, reflecting differential levels of gene exchange and potential local adaptation among populations in the Lüliang Mountain region.

### 3.6. PCoA Analysis Based on Microsatellite Markers

**Figure 3:**
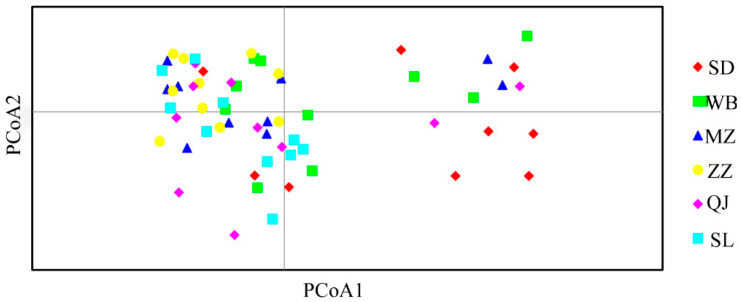
PCoA analysis of 60 colonies of *A. c. cerana* from 6 sample sites.

A two-dimensional principal coordinate analysis (PCoA) scatter plot was generated to assess the genetic relationships among populations (Figure 3). The results showed that most samples from Suide (SD) were distributed relatively distantly, indicating greater genetic divergence within this population. A few samples from Suide (SD), Wubu (WB), and Qingjian (QJ) exhibited scattered distribution patterns, suggesting partial differentiation. In contrast, samples from the other populations overlapped and clustered closely together, reflecting closer genetic relationships among those groups. This pattern is consistent with the genetic distance and similarity analyses, further confirming the reliability of the population structure assessment.

### 3.7. Clustering Analysis of Sampling Sites Based on Microsatellite Markers

**Figure 4:**
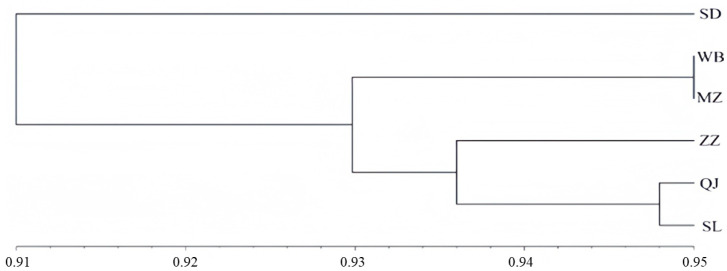
Cluster diagram of sample points.

Cluster analysis of the six sampling sites was performed using genetic similarity coefficients, and the grouping results are shown in Figure 4. The horizontal axis represents the confidence level of each cluster, while each branch corresponds to a group of genetically similar data points. Based on genetic similarity coefficients derived from microsatellite markers, the *A. c. cerana* populations from the Lüliang Mountains were divided into two major clades. The first clade consisted solely of the Suide (SD) population. The second clade was further subdivided into two branches: one comprising Qingjian (QJ) and Shilou (SL), which then clustered with Zizhou (ZZ), and the other comprising Wubu (WB) and Mizhi (MZ). This clustering pattern is largely consistent with the PCoA and genetic distance analyses, further supporting the robustness of the population structure assessment.

### 3.8. Genetic Diversity Analysis Based on Mitochondrial Sequencing

#### 3.8.1. Mitochondrial Sequence Electrophoresis

**Figure 5:**
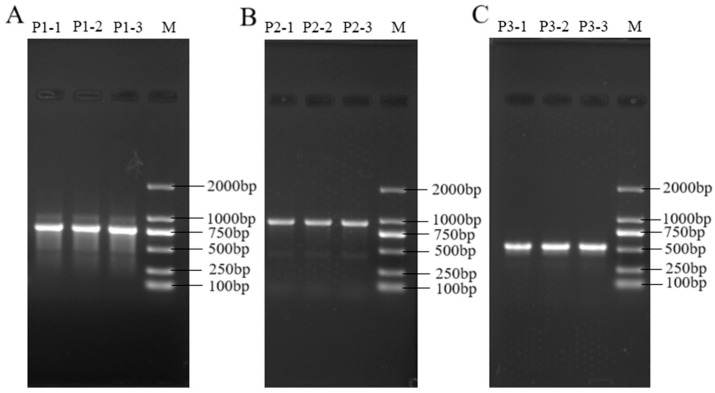
Mitochondrial PCR electrophoresis results. Note: (**A**) represents mtDNA COI~COII, (**B**) represents mtDNA COI, and (**C**) represents mtDNA Cytb, M represents Marker.

As shown in Figure 5, the amplification of mtDNA *COI–COII*, mtDNA *COI*, and mtDNA Cytb produced clear and single bright bands, indicating high specificity and suitability of the PCR products for subsequent experiments. This result further demonstrates that the primers used were well designed, with good specificity and amplification efficiency.

#### 3.8.2. Identification and Analysis of Nucleotide Variation Sites

Based on the amplification results of mtDNA COI–COII, mtDNA COI, and mtDNA Cytb from the six sampling sites, a total of 20 nucleotide variation sites were identified, leading to the construction of 19 haplotypes, as shown in Table 6.

#### 3.8.3. Distribution Characteristics and Frequency Analysis of Haplotypes

As shown in Table 7, among the 19 identified haplotypes, haplotype H4 was present across all sampling sites and was the most abundant, accounting for 30.0% of the total samples. The second most frequent haplotype, H3, was detected in seven samples, representing 11.67%, while haplotype H18 occurred in five samples, accounting for 8.33%. The remaining haplotypes were observed at relatively lower frequencies.

#### 3.8.4. Haplotype Diversity

Genetic diversity of *A. c. cerana* populations from the Lüliang Mountains was analyzed using DnaSP, and the results are summarized in Table 8. Overall, across the six sampling sites, the honeybee populations exhibited a haplotype diversity (Hd) of 0.884, a nucleotide diversity (π) of 0.00157, and an average number of nucleotide differences (K) of 3.144. At the individual sampling site level, haplotype diversity ranged from 0.356 to 0.889, nucleotide diversity from 0.00018 to 0.00252, and the average number of nucleotide differences from 0.356 to 5.044. The overall nucleotide base composition across all samples was A (35.4%), T (42.6%), C (12.5%), and G (9.4%).

A comparison of the six populations revealed that Zizhou (ZZ) showed the highest diversity, with a haplotype diversity of 0.889, an average nucleotide difference of 5.044.

## 4. Conclusions

This study provides the first systematic assessment of the genetic diversity and population structure of *A. c. cerana* in the Lüliang Mountains, thereby establishing a molecular foundation for the conservation and utilization of this regional honeybee resource. By screening 23 pairs of microsatellite loci (21 of which were polymorphic) and three mitochondrial DNA (mtDNA) fragments, we developed an efficient molecular marker system applicable to this area. Microsatellite analysis revealed a relatively high level of overall genetic diversity (mean PIC = 0.349), and observed heterozygosity (Ho = 0.827) exceeded expected heterozygosity (He = 0.608), resulting in a negative within-population inbreeding coefficient (FIS = −0.36). AMOVA partitioned 95.28% of genetic variation within populations and 4.72% among populations, corresponding to FST = 0.047. Gene flow (Nm = 2.74) was sufficiently high to maintain population connectivity. The overall inbreeding coefficient (FIT = 0.533) reflects population subdivision (Wahlund effect) rather than true inbreeding. This suggests that although differentiation has begun to emerge, relatively strong connectivity is still maintained across the Lüliang Mountain region. Mitochondrial DNA analysis further confirmed rich maternal genetic diversity, detecting 20 variable sites and constructing 19 haplotypes, with an overall haplotype diversity (Hd) of 0.884 and nucleotide diversity (π) of 0.00157. Both marker systems consistently showed that Zizhou and Mizhi represented the highest and lowest diversity sites, respectively, although the ranking order varied: microsatellite analysis indicated Qingjian > Wubu > Shilou > Suide > Zizhou > Mizhi, while mtDNA analysis indicated Zizhou > Shilou > Wubu > Qingjian > Suide > Mizhi. Genetic distance and clustering analyses revealed the greatest divergence between Suide and Zizhou (0.129), the closest relationship between Wubu and Mizhi (0.050), and an overall clustering pattern largely consistent with geographic distribution. Based on these findings, we recommend designating high-diversity areas such as Zizhou and Qingjian as core conservation zones, while standardizing colony introduction practices across regions to prevent excessive inbreeding and genetic erosion, thereby ensuring the sustainable utilization of *A. c. cerana* resources in the Lüliang Mountains.

## 5. Discussion

Microsatellite markers and mitochondrial DNA (mtDNA) remain the most powerful and complementary molecular tools for evaluating genetic diversity in honeybees and other livestock species. Microsatellites are widely applied because of their high polymorphism, co-dominant inheritance, and Mendelian segregation, allowing precise assessment of genetic variation, population structure, and gene flow. Previous studies have revealed distinct patterns of differentiation among *A. c. cerana* populations in different geographic regions. For instance, populations in the Wuyi Mountains exhibited significant genetic divergence due to isolation by high-elevation peaks [8]; colonies in Zhejiang Province showed marked morphological and genetic differences [9]; eastern Chinese populations displayed strong geographic isolation [10]; island populations in Hainan demonstrated high genetic diversity and clear separation from mainland groups [11]; and populations in the Changbai Mountains showed inter-site genetic variation [12]. These findings collectively indicate that environmental heterogeneity and geographic isolation play key roles in shaping the genetic structure of *A. c. cerana*. The combined use of microsatellite and mitochondrial markers provides a robust and efficient framework for characterizing honeybee population structure. Microsatellites capture contemporary gene flow because of their biparental inheritance and high mutation rates, whereas mtDNA traces maternal lineages and deeper evolutionary histories. Although whole-genome sequencing can generate high-resolution data, it remains cost- and computation-intensive for large population surveys such as the present study. The dual-marker strategy, therefore, offers a cost-effective and sufficiently informative alternative for conservation-focused analyses [13,14].

Mitochondrial DNA, owing to its maternal inheritance, high copy number, and moderate mutation rate, provides complementary insights into honeybee evolutionary history. Leelamanit [15] compared NADH dehydrogenase subunit 4 sequences among five Apis species from Japan, Nepal, and India, revealing substantial interspecific variation. Similarly, Henriques [16] analyzed over 1,000 colonies using the mtDNA COI–COII region and uncovered high maternal diversity across Portuguese populations of *Apis mellifera*. In this study, both microsatellite and mtDNA markers were applied to six *A. c. cerana* populations from the Lüliang Mountains, confirming that these two marker systems are indispensable and complementary for comprehensive genetic assessment.

Our results revealed that the expected heterozygosity (He = 0.608) of *A. c. cerana* in the Lüliang Mountains was higher than that reported for Hainan (He ≈ 0.049) and Zhejiang populations (He ≈ 0.3179) [17,18], but lower than that of the Qinba Mountains [19,20]. This suggests that the Qinba populations maintain higher polymorphism levels. Within the Lüliang region, He was lower than Ho (0.827), indicating a certain degree of differentiation and possible gene exchange among sampling sites. The microsatellite dataset revealed high levels of nuclear genetic variation across the Lüliang Mountain populations. Because observed heterozygosity consistently exceeded expected heterozygosity (Ho > He), the within-population inbreeding coefficient was negative (FIS = −0.36), reflecting heterozygote excess rather than inbreeding. AMOVA further showed that only 4.72% of total genetic variation occurred among populations, corresponding to FST = 0.047, a level considered low-to-moderate. The estimated gene flow (Nm = 2.74) was sufficiently high to counterbalance genetic drift and prevent strong population divergence. Importantly, the overall inbreeding coefficient of the pooled dataset (FIT = 0.533) reflects the Wahlund effect arising from subpopulation structure, rather than true biological inbreeding. These corrected hierarchical F-statistics consistently demonstrate that most genetic variation resides within populations and that gene exchange among sites remains frequent. Compared with populations from the Wuyi Mountains [21] and high-altitude regions of Longshan, Hunan [22], the Lüliang populations exhibited intermediate levels of differentiation. This is likely attributable to the geographical proximity of the six sites and the frequent exchange of colonies by local beekeepers, which promotes gene flow among neighboring populations.

Mitochondrial sequence analysis provided additional evidence regarding maternal lineages. Twenty polymorphic sites produced 19 haplotypes, with high overall haplotype (Hd = 0.884) and nucleotide diversity (π = 0.00157), both exceeding those reported in earlier nationwide assessments [23]. The Zizhou population exhibited the highest diversity, with the greatest number of variation sites (12, 60% of total) and haplotypes (6), while Mizhi had the lowest diversity, with only one variable site and two haplotypes. The universal presence of haplotype H4 across all populations suggests historical maternal gene flow, whereas site-specific haplotypes indicate ongoing localized differentiation. These findings demonstrate that the Lüliang Mountain region harbors rich *A. c. cerana* genetic resources with strong potential for breeding and conservation.

Differences between microsatellite and mtDNA patterns arise from their distinct inheritance modes, effective population sizes, and mutation rates. Microsatellites, with biparental inheritance and a larger effective population size, are sensitive to recent gene flow, explaining the elevated nuclear diversity detected in Qingjian. In contrast, mtDNA, which is maternally inherited and evolves more slowly, preserves historical maternal lineages and reveals the highest haplotype diversity in Zizhou. These complementary signals reflect evolutionary processes at different temporal scales and highlight the need to integrate both marker systems in conservation planning. Microsatellites capture nuclear genetic variation and reflect contemporary gene flow, whereas mtDNA traces maternal inheritance and historical lineage evolution. Differences between the two marker systems arise from contrasting inheritance modes, effective population sizes, mutation rates, and temporal resolution. Considering these complementary signals, both marker systems should be integrated when formulating conservation strategies for *A. c. cerana* in the Lüliang Mountain region. Therefore, comprehensive conservation strategies for *A. c. cerana* in the Lüliang Mountains should integrate both marker systems to obtain a multidimensional understanding of genetic diversity. Regions exhibiting high diversity, such as Zizhou and Qingjian, should be prioritized as core conservation zones. Furthermore, standardized management of colony introduction across regions is essential to minimize inbreeding and prevent genetic erosion, thereby ensuring the long-term sustainability of *A. c. cerana* genetic resources in northern China.

## Figures and Tables

**Table 1:** Primers for microsatellite labeling.

Sites	Forward Primer	Reverse Primer
BI216	TATCGTGATGGCGGATGC	TCCAATGATTATTTGGGCTCTC
BI278	AGGCCAACGTGCATGACG	GATGAGCAGCTAAGGTAACATCAGTATC
AP249	CGCGCGACGACGAAATGT	CAGTCCTTTGATTCGCGCTACC
SV220	TTTCTCGCGTAGAATGTAGAATAGG	AAGGATTTGCCTGCTACATGAC
AP066	TTGCATTCGGTCTCCAGC	ACTTGCCGCGGTATCTGA
SV261	ATCGTGTCCGACCAGTTCC	GCTAAATAGCTTGATTGCTCTCCT
AT185	CGCAGTGGAAATCATGGACG	CGGATAACCAGGGTTATGTAACG
BI225	GGTGCTTCACGCTTCTCGTAC	CGTTTCGGTGCGTATGTTG
AP208	GGCTTGTAAATTCGTGGAGG	CGAAACGGAAACTAGGCCT
AC139	ACCAGTGTTCACGGTAAACG	GATCATAGAGTACGCGCAAAG
AT103	CCTCCAATCGGCTAAACTCG	GCAGTCAGCGATCTCCAAGG
K0715	ACAGAAGCTCGAACACGATACC	AGTGGTCGATAACGCCGAG
AP148	GGAGCGAGGTGAACGACAC	GCCGGTAATTTCCAACCG
SV066	TTGCGCTAATGACTCGCG	CGTTTCCAAATGTGGTAAGTGGT
AT165	GCGACCACGTTTAACAGGAC	ACCAGTGAATTTGTTCATCGC
AP042	CGGATTAGGTTAGGTCGCG	GGCATACGTCCAACCCTGT
AT109	CGCGTTGCCAGACGTG	CGCAACCATCAAGATTCATC
AT101	GCGTTCCAAGTGAATGAACA	GTTGGCTATTTTCGTATCGC
UN117	TATCATACGCGCTTGATCCC	ATCCGGAGGGCCTGTGAC
K1458	ACCTCGATCCGTTCACACC	AGCTACGGGTGCTTTGTTCTC
UNEV2	AAGCGTCTGTAGGAAACACTGG	ACGGCAACTTGAGGTAAAGCT
UN270	GGAAAGCACAAACGATCGTG	CTCGAGCGTGCTTTGATGTAG
SV039	TTCCGCGGAAGATCTTCG	AAGAGACGCGCGAACGTC
Ap313	TAGCGCCCTAACGTCCAAC	CCCTTCTACCACCGACGC
AT004	TTCCACGGATGCACGGAC	TCCTTGCCCGCACAATCG
AC045	GATCGTAGTCGTGCAAAATAAGC	GTGTCCGTGATAACCGCAAC
AC011	CTTACGCCAATCTCTCCACG	CGGTTAATTTCGTTTCTCGC
AP189	TCCCACCTTCACCCTATCG	GCTTCTTTCCTTCTCGAGTCTC
BI314	GTATACAGAAACGCGACCAGG	GGATCATTTCTCCATCGAGG
Ap085	GATCAAACACACAAACGAAAGC	ACCGGAAGCCTAATCAAGG

**Table 2:** Mitochondrial sequencing primer information.

mtDNA Gene Fragment	Forward Primer	Reverse Primer
*COI~COII*	TCAGGGTATTCATAGGATC	CTATACCTCGACGATACTCAG
*COI*	CTCCAGATATAGCATTTCCTCG	TGCAAATACTGCTCCTATTGA
*Cytb*	GCTGCTGCATTTATAGGAT	AGACCAATTACTCCACCAAG

**Table 3:** Genetic diversity analysis of 6 sample sites.

Populations	PIC	Na (Observed No. of Alleles)	Ne (Effective No. of Alleles)	H (Nei’s Gene Diversity Index)	I (Shannon’s Information Index)	Ho (Observed Heterozygosity)	He (Expected Heterozygosity)
SD	0.294	2.652	1.816	0.336	0.596	0.852	0.645
WB	0.339	2.913	1.904	0.380	0.671	0.734	0.599
MZ	0.265	2.304	1.689	0.303	0.523	0.852	0.680
ZZ	0.287	2.478	1.762	0.352	0.560	0.843	0.657
QJ	0.341	2.652	1.907	0.394	0.677	0.839	0.585
SL	0.309	2.783	1.860	0.349	0.623	0.843	0.632
ALL	0.349	2.630	1.823	0.388	0.608	0.827	0.608

**Table 4:** Analysis of inter-site and intra-site molecular variance (AMOVA) of *A. c. cerana*.

Source of Variation	Degrees of Freedom	Sum of Square	Percentage of Variation (%)	*p* Value
Among pops	5	54.992	4.72	<0.05
With pops	114	480.6	95.28	
Total	119	535.592	100	

**Table 5:** Genetic similarity and genetic distance of 6 samples of *A. c. cerana*.

Locations	SD	WB	MZ	ZZ	QJ	SL
SD	****	0.924	0.924	0.878	0.917	0.908
WB	0.078	****	0.950	0.919	0.913	0.917
MZ	0.078	0.050	****	0.948	0.935	0.946
ZZ	0.129	0.084	0.052	****	0.927	0.947
QJ	0.086	0.090	0.066	0.075	****	0.948
SL	0.095	0.086	0.055	0.054	0.052	****

Note: Above the diagonal is genetic similarity, below the diagonal is genetic distance. Cells marked with “****” denote diagonal comparisons in which each population is compared with itself. Because self-comparisons yield fixed, non-informative values (genetic similarity = 1; genetic distance = 0), these entries are omitted and indicated with asterisks.

**Table 6:** Nucleotide variation sites.

	76	124	238	511	515	793	808	1037	1043	1116	1144	1187	1195	1247	1277	1427	1566	1722	1809	1926
H1	A	T	C	C	G	C	T	C	C	T	G	C	A	G	T	C	T	T	C	G
H2																		C		A
H3					A															
H4																				A
H5				T																
H6				T		T														
H7				T					T											
H8							C													A
H9												T								A
H10								T						A	C					
H11		C										T					A		T	
H12		C															A		T	
H13	G															T			T	A
H14	G							T								T			T	
H15											A									
16			T					T						A	C					
H17																	A		T	A
H18			T							A			T	A						
H19											A						A		T	

Note: The header represents 20 nucleotide variation sites, and H1–H19 are the constructed haplotype.

**Table 7:** Haplotype distribution.

Haplotype	SD	WB	MZ	ZZ	QJ	SL	Sample
H1	2					2	4 (6.67%)
H2	3						3 (5.00%)
H3	5					2	7 (11.67%)
H4		5	8		1	4	18 (30.00%)
H5		2					2 (3.33%)
H6		1					1 (1.67%)
H7		1					1 (1.67%)
H8		1					1 (1.67%)
H9			2				2 (3.33%)
H10				3			3 (1.67%)
H11				2			2 (3.33%)
H12				1			1 (1.67%)
H13				2			2 (3.33%)
H14				1			1 (1.67%)
H15				1			1 (1.67%)
H16					1		1 (1.67%)
H17					3	1	4 (6.67%)
H18					5		5 (8.33%)
H19						1	1 (1.67%)

**Table 8:** Genetic diversity.

Sampling Site	Sample Size (Number)	Polymorphic Sites (SNPs)	Number of Haplotypes	Haplotype Diversity (Hd)	Average Number of Nucleotide Differences (K)	Nucleotide Diversity (Pi)
OVERALL	60	20	19	0.884	3.144	0.00157
SD	10	3	3	0.689	1.489	0.00074
WB	10	5	5	0.756	1.667	0.00083
MZ	10	1	2	0.356	0.356	0.00018
ZZ	10	12	6	0.889	5.044	0.00253
QJ	10	9	4	0.711	4.044	0.00203
SL	10	5	5	0.822	1.822	0.00091

## Data Availability

The datasets generated and/or analyzed during the current study are available in the NCBI repository, https://www.ncbi.nlm.nih.gov/bioproject/PRJNA1289672 (accessed on 10 July 2025).

## References

[1] Li X., Ma W., Shen J., Long D., Feng Y., Su W., Xu K., Du Y., Jiang Y. (2019). Tolerance and response of two honeybee species *Apis cerana* and *Apis mellifera* to high temperature and relative humidity. PLoS ONE.

[2] Zhang Y., Xu H., Wang Z., Jie H., Gao F., Cai M., Wang K., Chen D., Guo R., Lin Z. (2023). A key gene for the climatic adaptation of *Apis cerana* populations in China according to selective sweep analysis. BMC Genom..

[3] Jara L., Muñoz I., Cepero A., Martín-Hernández R., Serrano J., Higes M., De la Rúa P. (2015). Stable genetic diversity despite parasite and pathogen spread in honey bee colonies. Naturwissenschaften.

[4] Chen S., Zhang Q., Chen Y., Zhou H., Xiang Y., Liu Z., Hou Y. (2023). Vegetation change and eco-environmental quality evaluation in the Loess Plateau of China from 2000 to 2020. Remote Sens..

[5] Chen L., Chai H., Du W., Lian Z., Wang Y., Li C., Cai L., Zhang L. (2025). Spatiotemporal dynamics of ecological quality and its drivers in Shanxi Province and its planned mining areas. Sci. Rep..

[6] Eimanifar A., Pieplow J.T., Asem A., Ellis J.D. (2020). Genetic diversity and population structure of two subspecies of western honey bees (*Apis mellifera* L.) in the Republic of South Africa as revealed by microsatellite genotyping. PeerJ.

[7] He J.M., Xu K., Du Y.L., Jiang H.B., Niu Q.S., Wang Z., Liu Y.L. (2024). Progress in research on mitochondrial genome of honey bees and their polymorphisms. J. Environ. Entomol..

[8] Liu M., Ji T., Yin L., Chen G. (2009). Relationship between microsatellite DNA markers and morphology feature of *Apis cerana cerana* populations in Wuyi Mountain. J. Fujian Agric. For. Univ. (Nat. Sci. Ed.).

[9] Zhao D.X., Su X.L., Cao L.F. (2013). Morphometric Characters of *Apis cerana cerana* in Zhejiang Province. Apic. China.

[10] Ji T., Yin L., Liu M., Chen G. (2009). Genetic diversity and genetic differentiation of six geographic populations of *Apis cerana* in East China. Acta Entomol. Sin..

[11] Xu X., Zhou S., Zhu X., Zhou B. (2013). Microsatellite DNA analysis of genetic diversity of *Apis cerana cerana* in Hainan Island, southern China. Acta Entomol. Sin..

[12] Yu Y.L., Zhou S.J., Xu X.J., Zhu X.J., Zhou B.F. (2013). Analysis on genetic diversity of *Apis cerana cerana* in Changbai Mountains. J. Fujian Agric. For. Univ. Nat. Sci. Ed..

[13] Harpur B.A., Minaei S., Kent C.F., Zayed A. (2012). Management increases genetic diversity of honey bees via admixture. Mol. Ecol..

[14] Meixner M.D., Pinto M.A., Bouga M., Kryger P., Ivanova E., Fuchs S. (2013). Standard methods for characterising subspecies and ecotypes of *Apis mellifera*. J. Apic. Res..

[15] Leelamanit W., Neelasaewee S., Boonyom R., Panyim S., Hayashi T., Yasue H., Amano K. (2004). The NADH Dehydrogenase Genes of *Apis mellifera*, *A. cerana*, *A. dorsata*, *A. laboriosa* and *A. florea*: Sequence Comparison and Genetic Diversity. J. Anim. Genet..

[16] Henriques D., Lopes A.R., Chejanovsky N., Dalmon A., Higes M., Jabal-Uriel C., Le Conte Y., Reyes-Carreno M., Soroker V., Martín-Hernández R. (2022). Mitochondrial and nuclear diversity of colonies of varying origins: Contrasting patterns inferred from the intergenic tRNAleu-cox2 region and immune SNPs. J. Apic. Res..

[17] Cao L.F., Lin R.P., Jiang Q.Q., Fu L.J. (2021). Monitoring on genetic diversity of Zhejiang Royal Jelly bee (Pinghu) using microsatellite loci and mitochondrial DNA. J. Zhejiang Univ. Agric. Life Sci..

[18] Cao L.F., Su X.L., Zhao D.X., Hua Q., Hu F.L. (2013). Genetic Diversity of Microsatellite DNA for *Apis cerana cerana* in Zhejiang. Apiculture of China.

[19] Guo H., Zhou S., Zhu X., Xu X., Yu Y., Yang K., Chen D., Zhou B. (2016). Population genetic analysis of *Apis cerana cerana* from the Qinling-Daba Mountain Areas based on microsatellite DNA. Acta Entomol. Sin..

[20] Li W.M. (2017). Genetic Diversity of Apis cerana in Qinling-Daba Mountain Area Based on Microsatellite and Mitochondrial DNA.

[21] Zhu X.J., Xu X.J., Zhou J.S. (2011). Genetic Analysis of *Apis cerana cerana* in Wuyi Mountain Nature Reserve Based on Microsatellite DNA. Fujian J. Agric. Sci..

[22] Xu H., Chen X.M., Lin Z.G. (2020). Microsatellite DNA analysis of the genetic diversity of *Apis cerana cerana* populations at different altitudes in Longshan, Hunan, central China. Acta Entomol. Sin..

[23] Ding G.L. (2006). A Study on Population Diversity of Apis cerana.

